# Our values and our historical understanding of psychiatrists

**DOI:** 10.1192/bjb.2023.16

**Published:** 2024-04

**Authors:** Claire Hilton

**Affiliations:** Royal College of Psychiatrists, London, UK; Birkbeck University of London, London, UK

**Keywords:** History of psychiatry, 20th century, ethics, eugenics, focal sepsis

## Abstract

Many people like to perceive themselves as better than previous generations: more knowledgeable, moral, tolerant and humane. Values associated with these aspects of ourselves may affect how we understand our professional forebears. In the early 20th century, some psychiatrists adopted new biomedical theories, including focal sepsis and eugenics, which resulted in inestimable harm. Detrimental clinical practices arose and were perpetuated in the context of societal values, medical ethics and other forces within and outside the medical profession. Historical understanding of the processes by which these things took place may help inform debate concerning current and future challenges of providing psychiatric care. The methods by which psychiatrists consider their predecessors may also have a bearing on how psychiatrists of the future will perceive us, the psychiatrists of the 2020s.

Many people, including in the medical profession, like to perceive themselves as better than previous generations: more knowledgeable, more moral, more tolerant and more humane. The assumption that we are better than our forebears may foster complacency about present-day psychiatry and discourage learning from our predecessors’ decisions and actions. In consequence, we may deprive ourselves of insights that could help inform our approach to challenges we encounter in the course of our work.^1^ As Peter Lepping and Rob Poole wrote recently: ‘The process of analysing and accepting psychiatry's past can help our profession to get closer to its real self and on a path to a better future’.^2^ This sense of self and identity with our forebears, as psychiatrists and through our professional institutions, can make balanced historical analyses all the harder.

The Royal College of Psychiatrists is one of many organisations seeking to understand its history without whitewashing the unfavourable. This task is far from straightforward. The College has recently debated whether we should ‘judge’ the past by past or present values.^3^ I would argue that although the present can assist us to probe the past, by providing new perspectives and tools to help us to formulate our questions about it,^4^ the present cannot provide a benchmark against which we can ‘judge’ our predecessors. If we use present-day values in this way, we create an uneven playing field whereby we disadvantage past generations, who could not have foreseen our frames of reference today.

The claim that we might critique our predecessors by current values and standards also assumes that we possess a single, uniform set of them. Political regimes and freedom of speech suggest otherwise. The ‘golden rule’, the ethical principle of treating others as one wants to be treated, found in most religions and cultures is also interpreted variably through legal, cultural, religious and other values-based frameworks that shape its practice. Consequently, ethics, values and behaviours today may differ between neighbours and colleagues on issues such as the sanctity of human life, including abortion and assisted dying. Rather than uniform, our values may be personal^5,6^ and hardly create a firm foundation for considering the actions of our predecessors.

Codes of medical ethics are also interpreted variably and in dialogue with the context of the time, including ideologies, science and other contemporary concerns. Regarding, for example, principles of beneficence and non-maleficence, some clinicians today acknowledge that medicine cannot be practised without harm, whether idiosyncratic reactions, side-effects or human error, when treating individual patients, or broader risks relating to environmental sustainability.^7,8^ Since medical ethical codes are contextualised, our predecessors’ words and actions would likewise have been determined by their own eras. Regarding psychiatry, it is also risky to consider its practice outside the legal frameworks that inevitably influenced past clinical decision-making, diagnosis and care: well into the 20th century in England, homosexual acts, attempts at suicide and admitting a person to a public mental hospital without a magistrate's order were all crimes.

As with all research, investigators need to adopt the most appropriate methodology to achieve the most meaningful answers: standard academic historical methods attempt to investigate the past from within it, as objectively as data will allow, rather than with hindsight. In our world of ‘woke’ awareness of racial prejudice and discrimination, culture, memory and commemoration, and the current and important widespread interest in historical rights and wrongs, we need to attempt to understand our forebears in their own setting. This essay explores aspects of this, not just who did what, but also how their actions came about and were promulgated. To do this, I have drawn on some controversial practices of early 20th century psychiatrists in the context of biomedical discoveries prominent at that time. I also consider how future generations may regard us, the psychiatrists of the turbulent early 2020s.

## Psychiatrists and the early 20th century biomedical context

Around the early 20th century, biomedical discoveries of invisible causes of disease, including micro-organisms, genetics, hormones and vitamins, plus new technologies, such as X-rays and blood transfusion, became established in medical practice. Psychiatrists, alongside their physician and surgeon colleagues, hoped that new discoveries would reveal aetiologies, prophylaxes and cures, especially concerning intractable and fatal disorders. Lacking rigorous codes of ethics regarding human experimentation, and with medical statistics in its infancy,^9^ medical practitioners in many specialties attempted new treatments compatible with the standards and knowledge of their day.^10^ Desperate diseases attracted desperate remedies, but reports by clinicians who introduced new treatments tended to magnify successes and obscure failures. This could perpetuate ineffective or detrimental practices.^11,12^

### Focal sepsis

Henry Cotton, psychiatrist and medical director of the State Mental Hospital at Trenton, New Jersey, sought to provide humane and ‘proper care of the insane’.^13^ He seized upon a widely accepted theory derived from microbiology: ‘focal sepsis’. Its proponents claimed that bacteria, their toxins or metabolic by-products in one organ of the body could cause damage in another. Against a background of knowledge that fevers caused delirium, poor oral hygiene was associated with endocarditis, and tetanus and diphtheria bacteria acted through toxins, it was not too far-fetched to suppose that similar mechanisms might cause mental disorders. Within the boundaries of accepted medical science, to eradicate focal sepsis, Cotton prescribed surgical removal of patients’ teeth and various organs. A complex multifaceted scenario ensued. This included external scientific reviews of Cotton's work which discredited it, indicating that his data were flawed and the surgery caused excessive harm. The generally well-respected Baltimore Professor of Psychiatry Adolph Meyer was involved in the analysis of the data, but there is evidence that he also helped conceal its conclusions, and Cotton continued with his surgical procedures.^14^ The combination of Cotton's hubris, the covering up of research findings and continuation of the surgical procedures with the approval of the institution's administrative leadership, showed a profound disregard of humanity both within and outside the medical profession.

Around the same time, medical practitioners other than psychiatrists were encouraging surgical procedures that aimed to eradicate focal sepsis from organs and tissues suspected of harbouring it. That included removing tonsils in which focal sepsis was assumed to be lurking, since the effects of focal sepsis were considered detrimental to children's physical and mental development. With the axiom of prevention being better than cure, ‘routine’ tonsillectomy became commonplace, acceptable to both public and professionals.^15^ In the UK in the 1920s, around 80 000 children underwent the procedure annually, and some died from it. Although Cotton is narrated as a historical pariah,^14,16^ the historiography of routine tonsillectomy largely lets its advocates off the hook.^17^ It would be surprising if the reality was quite so clear-cut. Rather, these contrasting scenarios suggest the need for honest and accurate historical contextualisation of past happenings and avoiding preconceived perspectives such as those of ‘anti-psychiatry’ or the ‘great men’ narratives common in the history of medicine more broadly. We need to explore the factors around when, how and why ‘the road to hell is paved with good intentions’ regarding patients’ lives.

### Eugenics

Another public and professional medical interest during the early 20th century was eugenics. Eugenicists, whose statistical methodology did not distinguish causal and associated factors, or nature and nurture, assumed the primacy of genetics for many disorders. They therefore aimed ‘to improve the biological quality of a population’ by influencing human reproduction through education, legal and physical means justified by their scientific knowledge.^18^ Concerning mental function, internationally, eugenicists proposed sterilising people with mental disability or chronic mental illness deemed to be inherited, to prevent similar conditions occurring in future generations. They also proposed sterilisation when they considered that the person's mental state made them incapable of bringing up their own children. However, medical, societal and governmental forces in different places created different practices. For example, in the USA, the state of Indiana passed the country's first eugenics-based sterilisation law in 1907.^19^ In the UK, a Sterilisation Bill proposed in Parliament in 1931 failed to become law.^20^ By contrast, in Sweden, from 1935 until 1975, the law allowed sterilisation on the advice of the medical profession and without the patient's consent.^21^

The scene was also different in Germany, where, in 1920, Professor of Psychiatry Alfred Hoche and Karl Binding, a lawyer, proposed ‘the destruction of life unworthy of life’ for people considered a burden on the state.^22^ Although debated, their call fell on fertile soil – including within psychiatry – and germinated in the context of social, political and economic turmoil after the First World War.^23^ In 1937, Ernst Rüdin, also a Professor of Psychiatry in Germany, addressed an international psychiatry congress about eugenics. He justified compulsory sterilisation for people with chronic mental conditions, since ‘The hereditary health of the people must come first and upon the mental specialist devolves an absolute duty to recognize this’. The audience opposed Rüdin with ‘unanimity of opinion’, citing scientific and societal arguments.^24^ Nevertheless, under the Nazi regime, Rüdin's scheme morphed into a process of annihilation, aligned with Hoche and Binding's ideas and to which other psychiatrists contributed.^25,26^

### Forces shaping clinical practice

In magnitude, the ultimate deleterious effects of Rüdin's and Cotton's actions are incomparable. However, for both, forces outside the medical profession influenced implementation of their methods, the state for Rüdin and the hospital authorities for Cotton. Also, both psychiatrists appeared to reject outright their peers who opposed them based on conflicting scientific evidence. The uncritical continuation of practices long after they have been shown to be detrimental is arguably more unethical than the misinformed but logically argued and seemingly well-intentioned introduction of new methods in the context of their times. Insulin coma treatment for schizophrenia provides another example of this: the practice continued, including in the UK, long after research indicated much harm with no benefit.^27–29^ How our forebears dealt with criticism and opposing evidence, and weighed up their theories and actions in the light of scientific and clinical observations,^30^ may give us pointers to an individual's medical ethical values, humility and humanity regarding clinical undertakings, contributing to how we should regard them today.

Other examples of societal and political forces shaping psychiatric practice included designating homosexuality as a mental disorder requiring ‘treatment’^31^ and the relationship between political dissidence and ‘sluggish schizophrenia’ in the former Soviet Union.^32^ Currently, in the USA, at the interface of psychiatry and physical medicine, we have the ‘opioid crisis’, attributed to the confluence of doctors’ efforts to improve pain management and aggressive marketing and pecuniary interests of the pharmaceutical industry.^33^ These episodes indicate the murkier side of medical practice, psychiatry in particular. Psychiatry, politics, law, science, ethics and societal values all interplayed in the past and continue to influence the realities of clinical practice. We ignore them at our peril.

## Conclusions: past, present and future

A short, history-based paper necessitates some degree of selection of illustrative examples. Those in this paper have been chosen to provoke thought rather than to be representative. Space does not allow further exploration of the complexities of the issues or the historical methodologies involved, but more details of these can be found in many of the references already cited. The situations discussed are extreme and, fortunately, rare but the principles and issues associated with them recur in less dramatic ways. Without historical understanding of how beneficial and detrimental clinical practices arose and were perpetuated, we will not learn from them in ways that could help us frame questions and inform debate, and generate and evaluate possible solutions concerning current challenges. It cannot be assumed that biomedical and other practices appropriate to clinical specialties in physical illness translate meaningfully into psychiatry, and just because practices become established does not mean that they are fit for purpose clinically, scientifically or ethically. That includes the current use of remote clinical consultations, appropriate for pandemic circumstances but requiring evaluation to ensure the greatest benefit and the least harm as circumstances change.^34^ A further current ethical consideration is the proposal for whole-genome sequencing of every UK newborn:^35^ reflection on the relationship between genetics and eugenics in the past may inform debate today on genetics and genetic engineering, alternatively known as ‘modern eugenics’.

Psychiatrists often generalise negatively about the practices of their forebears, many of whom, a century ago, worked in ‘asylums’, grappling with patients’ mental and physical disorders with inadequate illness classification systems, limited treatment options and constraints of under-resourcing, bureaucracy and a Lunacy Act unfit for purpose. Not all medical practice today is as good as it should be, and people suffer as a result. Every time we say ‘it is the best under the circumstances’ we imply that we are not treating our patients as we would wish.

The practice of psychiatry has always been complex and multifactorial, and understanding the past may help us better appreciate the diversity of perceptions and conceptions affecting contemporary, and future, goals. Future generations of psychiatrists, unless they understand the context and dilemmas of our clinical endeavours today, may well view us negatively as we might generalise about our predecessors, perpetuating the idea that they are better than their forebears and therefore writing off the possibility of learning from our mistakes alongside our successes. Hypothetically, future generations may one day write about us, the psychiatrists of the 2020s, that:

‘Morale in many of their multidisciplinary teams was dreadful, with high staff turnover. They admitted patients out of area miles from home. People with schizophrenia died years before their mentally well peers. The reality of care ‘in’ the community was care ‘by’ the community. They also kept changing their consensus-based, unscientific classifications of mental disorders. Much of what they did was wrong or harmful.’

These uncomplimentary perceptions might reflect the reality of practice for many National Health Service clinicians today, but they hardly reflect what we strive to achieve or the external constraints that gnaw at us, or how we try to overcome them. Future generations might be less critical if they adopt a nuanced, contextualised view of political, policy, economic, social, scientific, legal, COVID-19 and other influences on clinical services.^36^ For us today, by grasping the contextual elements, we are in the privileged position of being better able to understand and learn from our predecessors and, possibly, also to shape the history that future generations may write about us.

## Data Availability

Data availability is not applicable to this article as no new data were created or analysed in this study.

## About the author

**Claire Hilton**, FRCPsych, FRHistS, is historian in residence at the Royal College of Psychiatrists and an Honorary Research Fellow at Birkbeck University of London, London, UK.
